# Perspectives and challenges for mother-to-child transmission of HIV, hepatitis B, and syphilis in Brazil

**DOI:** 10.3389/fpubh.2023.1182386

**Published:** 2023-08-08

**Authors:** Angelica E. Miranda, Patricia C. Santos, Ronaldo A. Coelho, Ana Roberta P. Pascom, Leonor Henriette de Lannoy, Ana Cristina Garcia Ferreira, Pamela Cristina Gaspar, Ethel Leonor Maciel, Draurio Barreira, Gerson Fernando Mendes Pereira

**Affiliations:** ^1^Post-graduation Program in Infectious Diseases, Federal University of Espirito Santo, Vitória, Brazil; ^2^Department of HIV/AIDS, Tuberculosis, Viral Hepatitis and Sexually Transmitted Infections, Ministry of Health, Brasília, Brazil; ^3^Post-graduation Program in Public Health, University of Brasilia, Brasília, Brazil

**Keywords:** MTCT, HIV, syphilis, hepatitis B, pregnancy

## Abstract

**Background:**

Eliminating mother-to-child transmission (MTCT) of HIV, hepatitis B, and syphilis is a challenge in Brazil. Many policies have been implemented since 1986, but important gaps remain. This study aimed to describe the trends of MTCT in Brazil and evaluate the gaps and perspectives in this scenario.

**Methods:**

This is a descriptive study conducted with secondary data publicly available in the information systems of the Brazilian Ministry of Health regarding data on HIV, syphilis, and hepatitis B in pregnant women and children from 2011 to 2021.

**Results:**

HIV and hepatitis B have had constant rates over the years in pregnant women, with the detection rates around 2.5/1,000 live birth (LB) and 0.5/1.000LB, respectively. The same did not happen with syphilis, which has shown an increasing line in the last decade. In 2011, the detection rate of syphilis in pregnancy was 4.7/1,000LB, and in 2021 it reached 27.1/1,000LB. Regarding the trends in children, an important decrease was observed in HIV/AIDS (incidence rate from 0.18/1,000 in 2011 to 0.04/1,000 in 2021) and Hepatitis B (incidence rate from 0.9/1,000LB in 2011 to 0.5/1,000LB in 2021). For congenital syphilis, there is a continuous increase, being 3.3/1,000LB in 2011 and 9.9/1,000LB in 2021. Data from the HIV clinical monitoring showed that antiretroviral treatment coverage among pregnant women identified increased slightly between 2011 and 2021, in Brazil, from 92.3% to 94.3%. For syphilis, 82.5% of pregnant women were treated with benzathine penicillin, and 88.7% in 2011. The historical series of hepatitis B vaccination coverage in children has decreased over the years; it was 96% in 2013 and 76% in 2021.

**Conclusion:**

These data show many gaps and some perspectives in the MTCT program in Brazil. The country is close to reaching MTCT HIV elimination, but there are many challenges regarding HBV and syphilis. These data can be used to organize the strategies to improve the Brazilian response to MTCT elimination of HIV, hepatitis B, and syphilis.

## Background

The elimination of mother-to-child transmission (MTCT) of HIV, hepatitis B, and syphilis is an important challenge for the integrality of care, that is a result of the care offered to the user by multiple professionals, and expresses an indicator of the quality of services provided in the health care network (1). Prevention is possible with adequate diagnosis and treatment during prenatal care, childbirth, puerperium, or neonatal period (2). Achieving the goal of eliminating these conditions is a priority for the countries committed to the implementation of the 2030 Agenda to reach the Sustainable Development Goals (SDG), which was proposed by the United Nations (UN) for the elimination of important infections to public health by 2030 (3, 4).

Actions to prevent MTCT of HIV, syphilis, and viral hepatitis need to include a broad and comprehensive approach to women's health. Governments should think not only about the line of care during pregnancy but also about offering intervention opportunities throughout the life cycle of women (5, 6). Expanding access to diagnosis of these infections during prenatal care and adequate treatment to prevent MTCT should be priority measures in public health. Also, it is essential to be attentive to women's health beyond childbearing, which encompasses notions of empowerment, gender equality, and protection of human rights, including maternal health (2, 7).

Achieving the SDG goals is a priority for the Brazilian Ministry of Health (MoH) because MTCT of HIV, hepatitis B, and syphilis are public health problems in the country and are included in national policies (8, 9). Since 2017, Brazil has started to plan specific actions to approach the MTCT. A first national guide based on the WHO guidelines and focused on eliminating HIV was published in 2017. The document established indicators based on the WHO proposal and focused on a subnational elimination, so municipalities with 100,000 or more inhabitants could apply to be certified for the elimination of MTCT of HIV. In 2021, this document was updated to include syphilis and also criteria for tiers of good practices in health care to eliminate these infections. The tiers were proposed to recognize the achievements of countries as they progress along the path to elimination, and consider a set of criteria of good practice that include access to tests and treatment for the infections during antenatal care (10–12). The inclusion of hepatitis B MTCT elimination is planned for the 2023 process in Brazil.

Brazil presents inequalities and faces complex economic, social, and environmental transformations. Social, economic, and regional differences are still severe and extensive in the country; basic living conditions must be improved for a large part of the population; the Southeast and South regions are more populous and more developed, while the North and Northeast regions have a lower rate of access to health and a lower rate of urban development. Health problems are often a result of social and environmental variations and have remained persistent over the years (13). In recent years, the COVID-19 pandemic has put pressure on the public health system, evidenced its vulnerabilities, and contributed to the disruption of adequate antenatal and hospitalization care. The impact was heterogeneous across Brazil, depending on how local responses to COVID-19 and other health conditions (14).

Despite all the challenges, since 1989 the country has implemented many policies to control the MTCT of HIV, syphilis, and hepatitis B. The policies were focused on improving case surveillance, providing accurate tests to diagnose these infections, offering drugs and vaccines, and increasing access for the most vulnerable populations, among others (8). Box 1 summarizes the main Brazilian policies by year of implementation. Our goal in this study was to assess the trends of MTCT in Brazil in the last decade.

Box 1 **National policies to prevent MTCT of HIV, syphilis and hepatitis in Brazil.**

**Table:** 

**Year**	**Policies**
1986	Congenital syphilis became of compulsory notification, according to Ministerial Directive #542, 22 December 1986.
1992	HBV vaccine is offered to children under two years of age throughout the country.
1995	Based on PACTG 076 studies and national epidemiological data, the Ministry of Health of Brazil published the First Manual “General Considerations of the Binomial: HIV/Aids and pregnancy” with recommendations for vertical transmission prophylaxis.
1996	Unified Health System free distribution of antiretroviral medicines for people diagnosed with HIV and aids, according to the technical criteria established by Ministry of Health through the Law n° 9.313, 13 November 1996.
1997	Ministerial Directive #874 changed the use of AZT for women living with HIV during pregnancy and at delivery. The AZT became recommended for all immunological or clinical factors. This Directive also added AZT for neonates until the sixth week of life; combined therapy in pregnancy with protease inhibitor, and reinforced the contraindication of breastfeeding and its substitution for infantile formula.
1998	National Immunization Program recommends universal vaccination of children against hepatitis B from birth (first 12 to 24 hours)
1999	Viral Hepatitis B and C became of compulsory notification, according to Ministerial Directive #1.461, 22th December 1999.
2000	Manual of Reference Centers for Special Immunobiological – 2nd edition, 2000. Includes immunoglobulin for the prophylaxis of neonates born to mothers with hepatitis B.
2000	HIV during pregnancy and children exposed to HIV became of compulsory notification, according to Ministerial Directive #993/GM, 4 September 2000.
2002	Publication of the HIV mother-to-child Prophylaxis and Antiretroviral Therapy in Pregnancy manual (2002/2003) with orientations about the criteria for delivery vias selection and combined therapy for pregnancy, antiretroviral chemoprophylaxis at delivery and neonates, procedures for neonates at delivery, and assistance recommendation during puerperium.
2003	Updating of the congenital syphilis case definition for compulsory notification.
2004	National Study Sentinel-Parturient – HIV and syphilis
2005	Syphilis in pregnant women became of compulsory notification, according to Ministerial Directive #33, 14 July 2005.
2007	(1) Publishing the Operational Plan for HIV and Syphilis Vertical Transmission Reduction. (2) Publishing of the HIV and Syphilis Vertical Transmission Prevention Protocol.
2008	Publishing of the “How to prevent HIV and syphilis vertical transmission in your city” guide, in partnership with UNICEF.
2010	Acquired syphilis became of compulsory notification, according to Ministerial Directive #2472, 14 August 2010
2011	(1) Establishment of *Rede Cegonha* [Stork Network] for improving antenatal care. (2) HIV and syphilis rapid test implementation in primary healthcare units through *Rede Cegonha* [Stork Network], with federal, state, and municipalities sharing responsibilities, according to the Ministerial Directive #1.459 GM, 24 June 2011.
2013	Implementation of universal Test-and-treat approach to HIV/AIDS: recommendation for immediately starting the antiretroviral therapy after the HIV diagnosis in all immunological and virological conditions. Brazil was the first country in the World to implement the treatment as prevention in a public healthcare system. This initiative contributed substantially to decreasing the cases of HIV in children under five years old.
2014	(1) Based on PACTG 1043 study, Brazil updated the Clinical Protocol and Therapeutical Guidelines for HIV infection management in children and adolescents with dual chemoprophylaxis (oral AZT for four weeks associated with three doses of nevirapine) for neonates exposed to HIV from mothers that were not on antiretroviral therapy during pregnancy with documented viral load > 1.000 copies/mL at the last trimester of pregnancy. (2) Changing oral AZT recommendation from six to four weeks in the case of neonates from mothers on antiretroviral therapy during pregnancy. (3) Publishing of the HIV, Syphilis, and Viral Hepatitis Vertical Transmission Research Protocol, aiming to improve the surveillance, assistance, and management process for reducing vertical transmission cases.
2015	(1) Publishing of the first Clinical Protocol and Therapeutical Guidelines for Prevention of Vertical Transmission for HIV, Syphilis, and Viral Hepatitis, according to the Ministerial Directive #65, 9 November 2015. (2) Partnership with Federal Nursing Council (Cofen) to expand penicillin provision in primary health care, by the nursing staff, with the publication of Cofen Decision no. 0094/2015. (3) Decentralization of rapid syphilis tests for all primary health care units and maternity hospitals, besides the Rede Cegonha scope [Stork Network].
2016	(1) Partnership with Cofen for rapid HIV, syphilis, and viral hepatitis testing, with the publication of Cofen Decision no. 244/2016. (2) Release the syphilis indicators and basic data panel syphilis in pregnant women and congenital syphilis. (3) Purchase and distribution of benzathine penicillin in centralized mode by the Ministry of Health. (4) Launch the Strategic Actions Agenda for Syphilis Reduction in Brazil (2016–2017).
2017	(1) Publishing of the “Clinical Protocol and Therapeutical Guidelines for the Prevention of Vertical Transmission for HIV, Syphilis and Viral Hepatitis” 2nd edition. (2) Purchase and distribution of crystalline penicillin in centralized mode by the Ministry of Health. (3) Partnership with the Federal Council of Nurses (COFEN) for rapid reinforce the importance of penicillin prescription and administration by nurses, especially in primary health care units (Nota Técnica COFEN/CTLN N° 03/2017). (4) Establishment of the third October Saturday as the “National Day for Combating Syphilis and Congenital Syphilis”, a Bill approved by the House of Representatives, made official by Act no. 13.430 of March 31, 2017, sanctioned by the President of the Republic. (5) Launch the Strategic Actions Agenda for Syphilis Reduction in Brazil (2017–2019). (6) Publishing the Brazilian guidance on criteria and process for certification of Elimination of Mother-to-Child Transmission of HIV for municipalities with ≥ 100,000 inhabitants – 1st edition. (7) Launch the national intervention project “Syphilis No!” for Syphilis Reduction in Brazil.
2018	(1) Updating of the “How to prevent HIV and syphilis vertical transmission in your city” guide (partnership with the United Nations Children's Fund [UNICEF]). (2) Partnership with the Federal Council of Medicine (CFM) for rapid testing in pregnant women, with the publication of CFM Recommendation no. 1/2018. (3) Start of phase II study on cefixime's clinical efficacy to treat active syphilis in non-pregnant women in Brazil. (4) Combination of HIV and other STI Prevention: Brazil adopted new efficient technologies, such as Pre-Exposed Prophylaxis (PrEP) and Post-Exposed Prophylaxis (PEP). Related to vertical transmission, PrEP consists of one more prevention tool for the serum-discordant couple with children free of HIV. Additionally, PEP offers prophylaxis for children exposed to HIV through breastfeeding.
2019	(1) Agreement with partners to renew the “Strategic Actions Agenda for Syphilis Reduction in Brazil”. (2) Creation of HIV, syphilis, and Viral Hepatitis vertical transmission control group in the Ministry of Health (3) Online virtual national system (SIMC) to monitor public laboratory HIV-VL and CD4+ account data, integrated with the national ART dispensation system with a specific monitoring function for PW-HIV with detectable viral load (>50 copies/mL).
2020	(1) Serie of webinars about the Clinical Protocol and Therapeutical Guidelines for the Prevention of Vertical Transmission for HIV, Syphilis, and Viral Hepatitis. (2) Launch the Panel of indicators with the survey results conducted with maternity hospitals on actions for the Prevention of Vertical Transmission of HIV, Syphilis, and Viral Hepatitis. (3) Collaborative projects to encourage municipalities and states to eliminate vertical transmission of HIV and/or syphilis and to decrease STIs cases in vulnerable populations during the COVID-19 pandemic. (4) Launch the Strategic Actions Agenda for Syphilis Reduction in Brazil (2020–2021).
2021	(1) Publishing of the “Clinical Protocol and Therapeutical Guidelines for the Prevention of Vertical Transmission for HIV, Syphilis and Viral Hepatitis” 3rd edition. (2) Publishing flowchart for Birthing Centers and Hospital Maternity Services about the Prevention of Vertical Transmission for HIV, Syphilis, and Viral Hepatitis. (3) Launch the distance learning course free of charge and open access to the Prevention of Vertical Transmission for HIV, Syphilis, and Viral Hepatitis. (4) Nationwide Pilot implementation of CT/NG/TV/MG molecular detection and Dual HIV/syphilis rapid diagnostic tests in antenatal care services. (5) Publishing the 2^nd^ version of the Brazilian guidance on criteria and process for certification of Elimination of mother-to-child Transmission of HIV and Syphilis in municipalities with ≥ 100,000 inhabitants. In this 2^nd^ version, municipalities with ≥ 100,000 inhabitants making progress toward elimination could obtain certification of their progress through the Path to Elimination (PTE) and its three levels of achievement – bronze, silver, and gold.
2022	(1) Publishing the recommendations for using dolutegravir in pregnant women regardless of gestational age and for women living with HIV of childbearing age aiming to get pregnant. (MoH Decree #1/2022-CGIST/DCCI/SVS/MS). (2) Publishing the updated HIV, Syphilis, and Viral Hepatitis Vertical Transmission Research Protocol. (3) Launch the National Agreement to eliminate mother-to-child transmission of HIV, Syphilis, Hepatitis B, and Chagas Disease. (4) Certification of eliminating vertical transmission of HIV and/or syphilis or seal of good practices for 43 with ≥ 100,000 inhabitants. (5) Launch the epidemiological panel with Nationwide mother-to-child transmission of HIV and/or Syphilis data for healthcare managers. (6) Publishing the final report with the survey results conducted with maternity hospitals on actions for the Prevention of Vertical Transmission of HIV, Syphilis, and Viral Hepatitis. (7) Increase the discussion about the certification process for the Elimination of mother-to-child Transmission of Viral Hepatitis and Chagas.

## Methods

This is a descriptive study conducted with secondary data publicly available in the information systems of the Brazilian Ministry of Health regarding data on HIV, syphilis, and hepatitis B among pregnant women and children from 2011 to 2021.

The indicators analyzed were obtained from the data available in the panel of indicators of the Department of Sexually Transmitted Infections, Tuberculosis, HIV/AIDS and Viral Hepatitis at the Health Surveillance Secretary of the Ministry of Health of Brazil (DVIAHV/HHS/MoH). The panel of indicators generates data from the national notifiable diseases information system, as known as SINAN. The information of this system is originated from the Individual Notification Form (INF), which is completed electronically by primary care unit professionals for each patient when there is suspicion of a health problem that requires compulsory notification. The INF is forwarded to the services responsible for epidemiological surveillance in the Municipal Health Secretariats, which must weekly transfer the files to the State Health Secretariats. After review in the states, data are sent electronically to the Ministry of Health, which is responsible for consolidating and publishing national data.

The following indicators were analyzed: (1) detection rates of HIV in pregnancy and children identified in one of the HIV databases (treatment and CD4 or viral load exams) (http://indicadores.aids.gov.br); (2) antiretroviral (ART) coverage among pregnant women (http://indicadoresgestantes.aids.gov.br/); (3) syphilis in pregnancy and congenital syphilis (http://indicadoressifilis.aids.gov.br); (4) detection rates of hepatitis B in pregnancy and in children (http://indicadoreshepatites.aids.gov.br/); and (5) Hepatitis B vaccination within 30 days of birth and the pentavalent (Diphtheria, Pertussis, Tetanus, Hepatitis B and Hib) coverage (http://tabnet.datasus.gov.br/cgi/dhdat.exe?bd_pni/cpnibr.def).

The detection rates of HIV and hepatitis B in children were presented by year of birth; the incidence of congenital syphilis was presented by year of diagnosis. The detection rates of pregnant women with syphilis, HIV, and HBV and the incidence of congenital syphilis and the detection rate of HBV in children under 1 year and AIDS in children under 5 years of age, and HIV infection in children under 1 year of age were calculated over the historical series from 2011 to 2021. The vertical HIV transmission rate, which is the ratio between exposed and infected children, was calculated from the number of children infected with HIV per year of birth divided by the number of pregnant women notified according to the year of delivery, taking this information as a proxy for the total number of exposed children.

Data collected from the panel of indicators were included in a spreadsheet in the Statistical Package for the Social Sciences - SPSS 20.0 software, where they were verified to exclude duplication and prepared for analyzing the variables included in the study. These data were analyzed descriptively using the SPSS 20.0 software tools. Descriptive analysis was performed, including frequency distribution for qualitative variables, and appropriate graphics were developed.

As the analyzed data were publicly available, the study was dispensed by the evaluation by the ethics committee. Still, it was conducted in agreement with the National Ethics Committee #466 from December 12, 2012.

## Results

During the study period, 85,470 pregnant women were notified with HIV, 466.584 with syphilis, and 15,411 with HBV. Regarding children, 3,781 were notified with HIV, 221,600 with congenital syphilis, and 1,196 with HBV.

**Figure 1:**
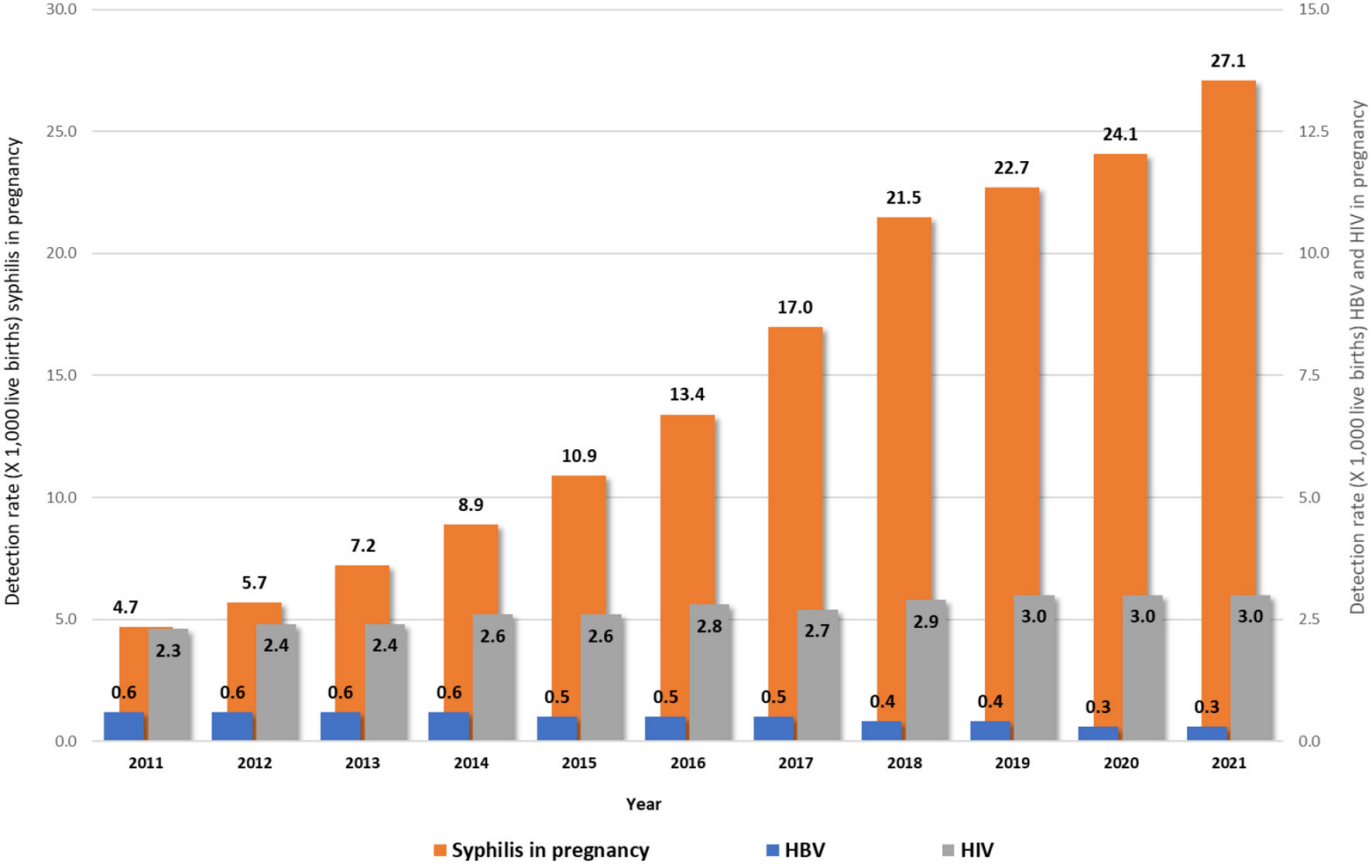
Rates of HIV^1^, syphilis^1^, and HBV^2^ notifications in pregnant women in Brazil, 2011–2021. Source: DVIAHV/HHS/MoH. (1) Cases reported on Sinan by 06/30/2022. (2) Cases reported on Sinan by 12/31/2021.

Figure 1 shows the rates of HIV, syphilis, and HBV notifications among pregnant women, in Brazil, from 2011 to 2021. It can be observed that trends of HIV and hepatitis B have remained constant over the years. The same did not happen with syphilis, which has shown an increasing line in the last decade. Regarding hepatitis B, available information indicated that 10.2% from all 150,761 notified cases occurred in pregnant women from 2011 to 2021.

**Figure 2:**
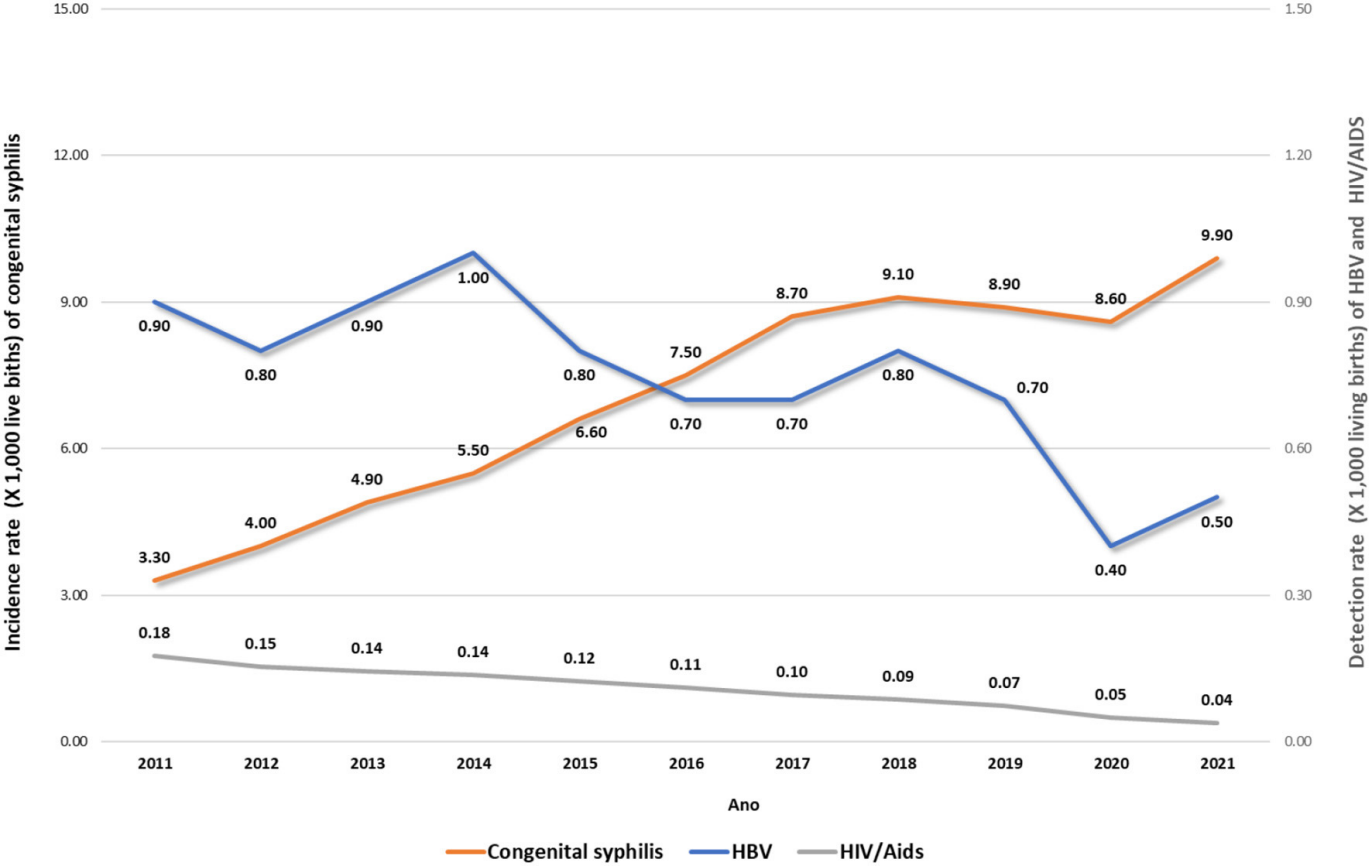
Rates of HIV/AIDS^1^, syphilis^1^, and HBV^2^ notifications in children, by year of birth. Brazil, 2011–2021. Source: DVIAHV/HHS/MoH. (1) Cases reported on Sinan by 06/30/2022. (2) Cases reported on Sinan by 12/31/2021.

The rates of HIV, syphilis, and HBV notifications in children in Brazil from 2011 to 2021 are presented in Figure 2. Hepatitis rates in children remained low during the analyzed periods, and HIV infections have been falling over the years. However, syphilis rates have remained high in the last decade and kept increasing.

**Figure 3:**
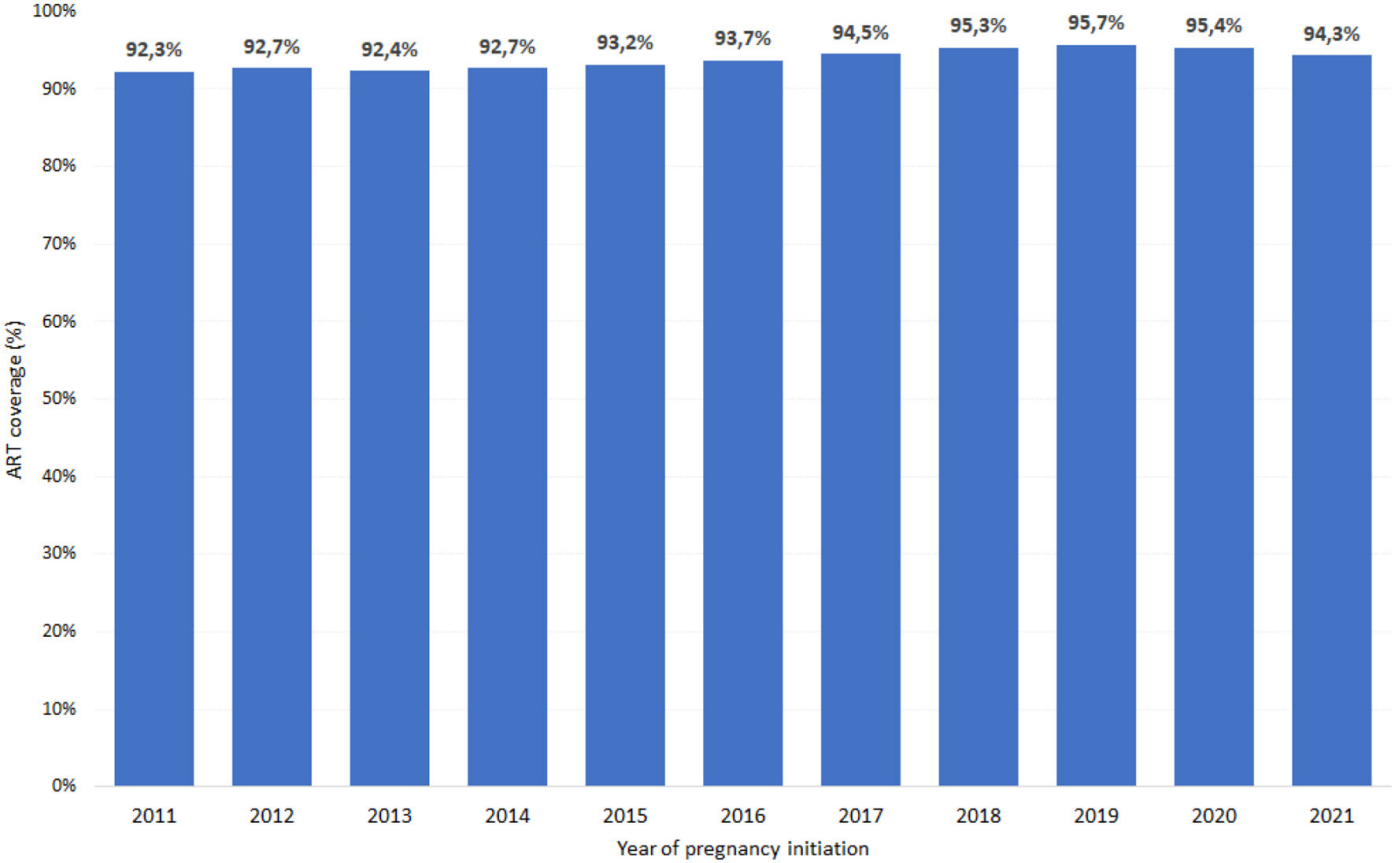
Proportion of antiretroviral treatment coverage among pregnant women by year. Brazil, 2011–2021. Source: DVIAHV/HHS/MoH.

Figure 3 shows that the antiretroviral treatment coverage among pregnant women identified increased slightly between 2011 and 2021, in Brazil, from 92% to 94%. It can be observed that the proportion is stable over the years.

**Figure 4:**
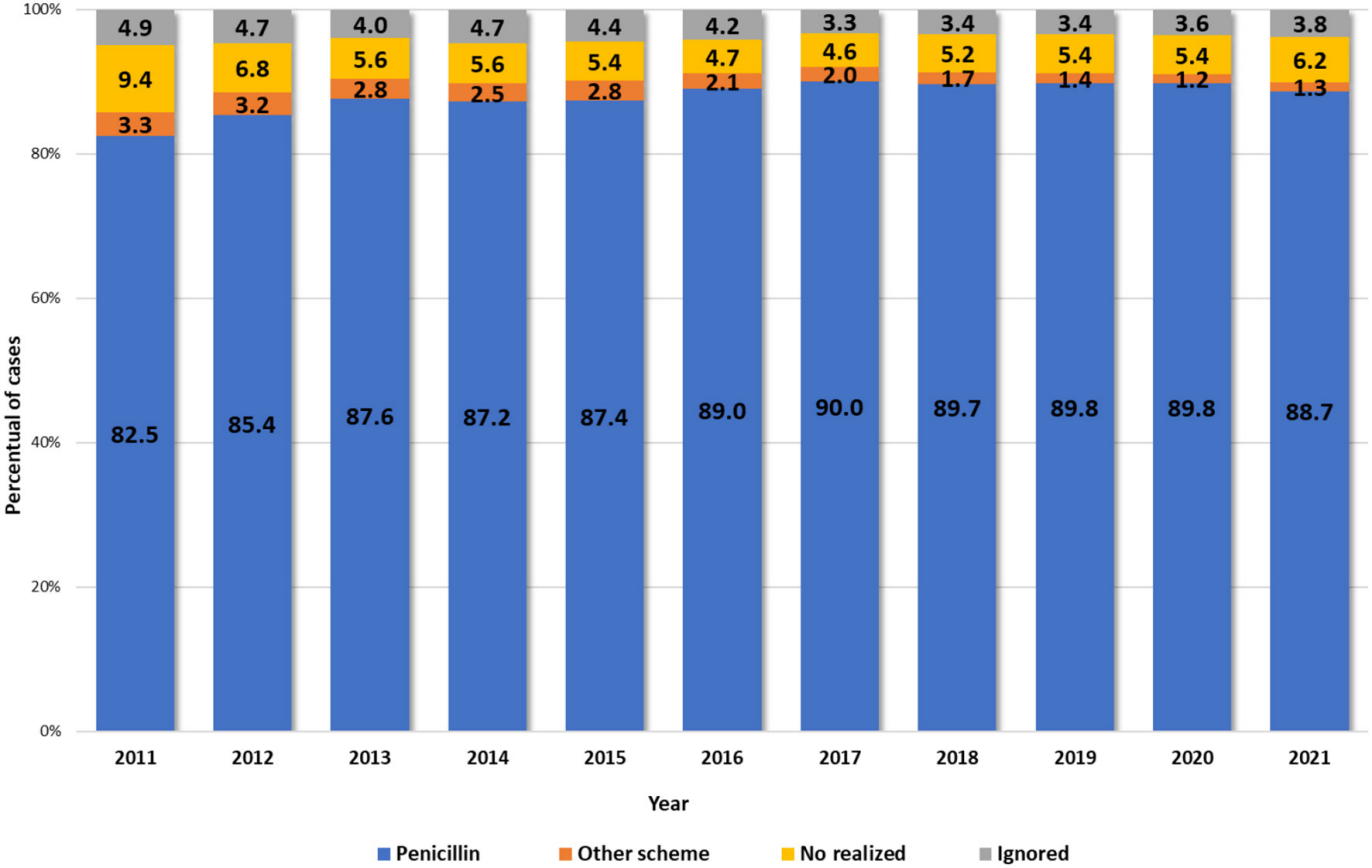
Distribution of cases proportion^1^ of pregnant women with syphilis according to the prescribed treatment regimen^2^ by year of diagnosis. Brazil, 2011 to 2021. Source: DVIAHV/HHS/MoH. (1) Cases reported on Sinan by 06/30/2022. (2) Prescribed treatment with at least one dose of benzathine penicillin, regardless of the clinical form.

The historical series of the proportion of cases of pregnant women with syphilis according to the prescribed treatment regimen in Brazil from 2011 to 2021 are described in Figure 4. Almost 90% are adequality treated using Benzathine Penicillin, but there is still concern about the fact that 6.2% did not undergo treatment during pregnancy in 2021.

**Figure 5:**
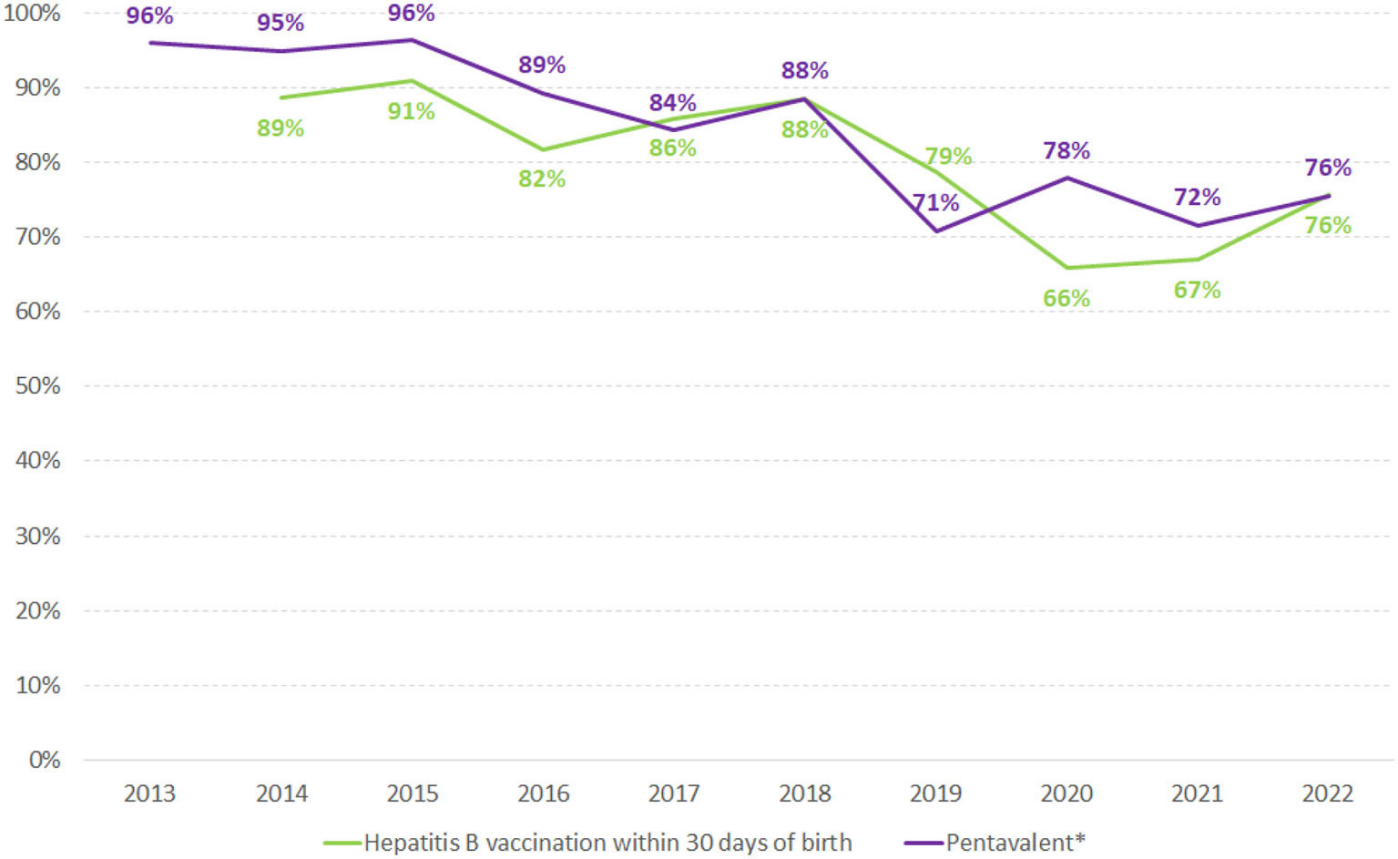
Proportion of hepatitis B vaccination within 30 days of birth and pentavalent* vaccination in children in Brazil, 2013–2021. Source: National Immunization Program/HSS/MoH. (*) Pentavalent vaccine includes Diphtheria, Pertussis, Tetanus, Hepatitis B, and Hib.

The historical series of the proportion of hepatitis B and pentavalent vaccination coverage in children are described in Figure 5. The universal offer and notification of these data started in 2013 and 2014. A drop in the proportion of vaccinated children over the years can be observed, despite the availability of vaccines in the national immunization program.

## Discussion

These data can help assess the aspects related to MTCT prevention of HIV, syphilis, and hepatitis B in Brazil, and will facilitate the strategic planning to enable the progress of the Brazilian response to MTCT elimination of these infections. The results point out that Brazil is approaching the WHO rate of HIV MTCT elimination as a public health problem and still needs to improve strategies for eliminating syphilis and viral hepatitis MTCT. As the strengths of these policies, Brazil has a universal public health system, the SUS, and a strong STI/AIDS program that includes free counseling, vaccines for HBV, diagnostic tests and treatment for HIV, hepatitis B, and syphilis, lactation inhibitors and infant milk formula, MTCT guidelines, and national health information systems (12, 15–17).

However, to achieve the SDG, health surveillance processes must be integrated with current strategies to strengthen Primary Health Care (PHC). A permanent effort is needed at the tripartite government level, involving federal, state, and municipal management for the success of the strategies, the incorporation of technologies, the permanent education of health workers, and the qualification of care and health surveillance actions. From a broad perspective, it also seeks to articulate health workers, teaching and research institutions, public and private health services, and civil society, mobilizing engagement in the implementation, monitoring, and dissemination of these actions (17–19).

The country has faced many challenges regarding the prevention of MTCT of HIV and syphilis and is now approaching a new one, which is to include HBV in the process (20–22). The identified weaknesses are the large geographic dimensions, demography, regional sociocultural diversity, overload of the public health system, the volatility of the technical team attending at the primary health care units, and clinical monitoring for the exposed and infected children (17, 23). These situations can be considered threats to the process. Also, it can be cited the completeness of the information data systems, such as inconsistencies, duplicities, and missing cases that happens because of the enormous amount of data and three management instances (municipal, state, and federal), data verification, which is a complex process (24, 25).

It is also worth mentioning that HBV notification is unique and occurs at the time of diagnosis of the infection. It is essential to point out that there is no specific form for notifying pregnant women with hepatitis B in Brazil, which causes difficulties in knowing the real number. It is known that from all notified cases, around 10% occurred in pregnant women from 2000 to 2021. Children exposed to hepatitis B are also not notified, as there is still no complete implementation of surveillance of vertical transmission of hepatitis B in the country (26). Notification data on HBV vaccination show a decrease in the country, mainly regarding the vaccination in the first 30 days of birth. Unpublish data reported that this data could be underestimated because the maternity hospitals give the vaccine but do not register it because of problematic communication with the national vaccination information system. A more detailed investigation will be important to elucidate this information.

Although, different opportunities can be identified: the Pan American Health Organization supports Member States to commit to the elimination of MTCT of HIV, syphilis, hepatitis B, and Chagas disease in the Region (10); the national visibility of the problem in Brazil, multisectoral approach, and availability of political interest on MTCT control (12, 20). Among the opportunities to succeed in this process, the SUS makes available the necessary inputs to prevent vertical transmissions free of charge. There are official documents that regulate the quality of care, develop the training of multidisciplinary teams in maternity hospitals and improve the quality of care for pregnant women, women in labor, and newborns, to reduce vertical transmission of HIV and control congenital syphilis (9). The proposal is to share responsibilities with states and municipalities and targets for the staggered and regionalized reduction of vertical transmission rates.

This process has many threats, such as the quality of prenatal care, which sometimes misses opportunities to test and treat the pregnant. Access to prenatal care needs to be improved to increase the inclusion of the most vulnerable people and reduce stigma and discrimination, complicating access. In addition, the training processes of the health professional team still need to be improved and often show regional differences in a country of continental dimensions (27, 28).

Among the limitations of this study, it can be mentioned the use of secondary data without independent validation, data coming from the health administration, and subject to information bias. Another limitation comes from the design of an ecological study, which does not allow direct interpretation of the results at the individual level. The descriptive approach is limited to univariate analyses, not adjusting for different risk factors and their interactions, nor for the spatial structure of data, which would be possible in a more complex analytical approach. However, these infections are of compulsory notification, and the information systems are the most comprehensive data sources available to carry out this evaluation. Therefore, the carried-out analyses allow for a distribution of possible underreporting of cases to be diluted in a less heterogeneous way. In addition, these diseases participate in national agreements, which involve the rendering of accounts and financing for states and municipalities. Good data coverage is expected, allowing extrapolation to the total population.

## Conclusion

These data indicate that there is still a considerable distance to cover in order to eliminate MTCT in Brazil. Nevertheless, the collaborative efforts across various sectors represent a significant step forward in this regard. Moreover, since the implementation of the Sanitary Reform in Brazil, the country has made substantial progress toward achieving universal, equitable, and comprehensive access to healthcare services. There has also been a commitment to aligning with global recommendations to promote the surveillance, prevention, and control of MTCT since the 1990s. These initiatives offer invaluable tools for a collective national endeavor.

The certification of subnational elimination of MTCT of HIV and syphilis has emerged as a strategic approach recommended by the Ministry of Health to streamline the healthcare network. It has played a crucial role in enhancing preventive measures, diagnostics, care, and treatment for pregnant women and their infants, while also improving the quality of epidemiological surveillance. This continuous certification process has been developed in collaboration with state and municipal authorities. In addition to the certification itself, this process has strengthened the management and care network for maternal and child health in the Brazilian public health system and improved the quality of care.

## Data availability statement

The datasets presented in this study can be found in online repositories. The names of the repository/repositories and accession number(s) can be found in the article/supplementary material.

## Author contributions

AM: conceptualization, formal analysis, investigation, methodology, supervision, validation, writing original draft, and final writing (review and editing). PS, RC, and AP: conceptualization, data curation, formal analysis, methodology, software analysis, validation, and final writing (review and editing). LL: conceptualization, data curation, formal analysis, methodology, validation, and final writing (review and editing). AF and PG: investigation, methodology, validation, writing original draft, and final writing (review and editing). EM and DB: investigation, methodology, validation, and final writing (review and editing). GP: investigation, methodology, validation, writing original draft, and final writing (review and editing). All authors contributed to the article and approved the submitted version.
